# Alkali and alkaline earth elements in follicular fluid and the likelihood of diminished ovarian reserve in reproductive-aged women: a case‒control study

**DOI:** 10.1186/s13048-024-01414-3

**Published:** 2024-05-18

**Authors:** Tian Tian, Qin Li, Fang Liu, Huahua Jiang, Rui Yang, Yue Zhao, Fei Kong, Yuanyuan Wang, Xiaoyu Long, Jie Qiao

**Affiliations:** 1 Center for Reproductive Medicine, Department of Obstetrics and Gynecology, Peking University Third Hospital, 49 North Garden Rd, Haidian District, Beijing, 100191 China; 2 National Clinical Research Center for Obstetrics and Gynecology, Peking University Third Hospital), Beijing, China; 3 Key Laboratory of Assisted Reproduction (Peking University), Ministry of Education, Beijing, China; 4 Beijing Key Laboratory of Reproductive Endocrinology and Assisted Reproductive Technology, Peking University Third Hospital), Beijing, China; 5 Department of Maternal and Child Health, School of Public Health, Peking University, Beijing, China; 6 Beijing Advanced Innovation Center for Genomics, Beijing, China; 7 Peking-Tsinghua Center for Life Sciences, Peking University, Beijing, China

**Keywords:** Diminished ovarian reserve, Alkali elements, Alkaline earth elements, Follicular fluid, Female fertility, Bayesian kernel machine regression (BKMR)

## Abstract

**Background:**

Imbalances in alkali elements (AEs) and alkaline earth elements (AEEs) cause reproductive disorders. However, it remains unclear whether AEs/AEEs in follicular fluid have a relationship with the serious reproductive disorder known as diminished ovarian reserve (DOR).

**Methods:**

A nested case‒control study was carried out in China. Follicular fluid samples from 154 DOR patients and 154 controls were collected and assessed for nine AEs/AEE levels. Both the mixed and single effects of the elements on DOR were estimated with a Bayesian kernel machine (BKMR) and logistic regressions.

**Results:**

The DOR group had higher median concentrations of Li, Na, and K in follicular fluid (all P values < 0.05). The logistic regression showed that compared with their lowest tertile, the high tertiles of K [OR:2.45 (1.67–4.43)], Li [OR: 1.89 (1.06–3.42)], and Cs [OR: 1.97 (1.10–3.54)] were significantly associated with the odds of DOR. The BKMR model reported that the DOR likelihood increased linearly across the 25th through 75th percentiles of the nine-AE/AEE mixture, while the AE group contributed more to the overall effect.

**Conclusion:**

This study revealed an association in which the likelihood of DOR increased with higher overall concentrations of AE/AEEs in follicular fluid. Among the nine detected elements, K, Li, and Cs exhibited significant individual associations with DOR. We provide new clues for the environmental factors on female fertility decline.

**Trial registration:**

Retrospectively registered.

**Supplementary Information:**

The online version contains supplementary material available at 10.1186/s13048-024-01414-3.

## Introduction

Infertility is estimated to affect 10–15% of reproductive-aged couples worldwide [1]. In countries with rapid social and economic development, an increasing number of women are postponing childbearing as they pursue their studies and careers, and the concomitant decrease in fecundity and infertility has aroused great concern [2, 3]. For example, the prevalence of infertility in China increased from 11.9% in 2007 to 18.7% in 2018 [4]. The decline in both quantity and quality of oocytes is one of the fundamental reasons for fertility disorders in women, clinically defined in reproductive medicine as diminished ovarian reserve (DOR) [5, 6]. The role of female age is widely recognized as a crucial factor in DOR [6]. Nonetheless, it’s important to note that women of identical age can exhibit substantial variations in ovarian reserve [7]. Moreover, there is a trend of DOR affecting women at increasingly younger ages, suggesting that factors beyond chronological age may affect DOR [8, 9]. Environmental exposures have emerged as potential contributors to the development of DOR, indicating their potentially significant role [10–15].

Among the various environmental factors, elements such as the alkali elements (AEs), including lithium (Li), sodium (Na), potassium (K), rubidium (Rb), cesium (Cs), and francium (Fr), and the alkaline earth elements (AEEs), including calcium (Ca), magnesium (Mg), strontium (Sr), barium (Ba), beryllium (Be), and radium (Ra), are ubiquitous and play vital roles in physiological and pathological processes in humans [16]. In the AE group, K, Na, and Li are the most abundant physiological metal ions in living organisms [17, 18]. Na/K channels play crucial roles in biological processes [16]. Notably, one study reported that increased sodium intake from food was associated with polycystic ovary syndrome [19]. Other AEs, such as Rb and Cs, may substitute for K in the body, but the biological functions of Rb and Cs remain unclear [16]. Regarding AEEs, clinical trials have shown that supplementation with Cs can influence the ovarian response during the IVF process [20] and can affect on pregnancy and live birth rates [21, 22]. A higher blood Ba concentration has been reported to be associated with a greater risk for polycystic ovarian syndrome (PCOS) [23]. The findings of these studies suggest the potentially important role of AE and AEEs in female reproductive health.

To date, few studies have reported direct associations between the AE/AEE concentration and ovarian reserve, the cornerstone of female fecundity. For oocytes, their development and maturation occur within a barrier environment composed of follicular fluid formed by granulosa cells and plasma ultrafiltrate, without direct contact with the bloodstream [24, 25]. Therefore, analysis of follicular fluid is recognized as providing a more accurate estimate of exposures that may influence reproductive outcomes, as the fluid closely mirrors the microenvironment surrounding the developing oocyte that is particularly pertinent to disorders of oocyte development (DOR). In addition, based on a review of the literature, previous studies have focused only on the individual effects of environmental elements in serum on infertility and other reproductive diseases [26]. However, in daily life, humans are inevitably exposed to a series of elements simultaneously. Animal and human studies have suggested that mixtures of multiple pollutants could have more significant impacts on health outcomes than the individual effects of each chemical [27, 28]. Exploring the combined effect of AEs and AEEs can better reflect the human exposure route and provide novel clues for the association between environmental factors and female fertility.

Therefore, in this study, we aimed to investigate whether both single and mixed exposure to AE and AEEs via follicular fluid are associated with the likelihood of DOR. To investigate this possibility, we performed a nested case‒control study including 154 DOR women and 154 women with a normal ovarian reserve. Concentrations of nine AE/AEEs in the follicular fluid of the women were assessed, and both the mixed and single effects of the detected elements on DOR were analyzed.

## Materials and methods

### Study design and participants

This study is based on a nested case‒control design. We selected participants from the prospective clinical cohort of patients who underwent in vitro fertilization/intracytoplasmic sperm injection (IVF/ICSI) treatment at the Centre of Reproductive Medicine, Peking University Third Hospital in China. Women who met two of the following three criteria would be defined as having DOR and included in the case group: (i) a bilateral antral follicle count (AFC) ≤ 5, (ii) a serum anti-Müllerian hormone (AMH) level of ≤1.1 ng/ml, and (iii) a basal serum follicle-stimulating hormone (FSH) level of ≥10 IU/L on the second or third day of the menstrual cycle [29]. Women with a normal ovarian reserve (who were diagnosed with AMH, FSH, and AFC levels) and who underwent IVF/ICSI treatment due to male infertility composed the control group. To control for the potential confounding effect of age on ovarian reserve, we matched the case and control groups at a 1:1 ratio of age (± 1 year). Women with other disorders affecting ovarian reserve, including genetic disorders, polycystic ovaries, PCOS, a history of ovarian surgery, and endometriosis, or who took hormone drugs within six months before the hormone test were excluded from the present study. A total of 308 participants (154 DORs vs. 154 controls) aged 20–40 years between October 2020 and July 2022 were included in the study.

Written informed consent was obtained from all participants. This study was approved by the ethical committee of the institutional review board of Peking University Third Hospital (M2021431).

### Data collection

The basic information included sociodemographic variables (including female and male age, ethnicity, occupation, educational levels, and parity), body mass index (BMI), history of disease, the type of infertility, reproductive hormones [including follicle-stimulating hormone (FSH), estradiol (E2), progesterone (P), luteinizing hormone (LH), testosterone (T), prolactin (PRL), and anti-Mullerian hormone (AMH)], and variables related to the treatment process collected from the clinical cohort database. When the basic examination of a participant was finished, and before the ovulation day, a trained healthcare worker performed a face-to-face interview using a structured questionnaire to collect environment-related information, such as smoking and drinking information.

### Sample collection

The follicular fluid samples were obtained by experienced nurses based on the process of oocyte retrieval. On the day of oocyte retrieval, follicular fluid was collected in sterile Petri dishes using follicle aspiration under ultrasonic monitoring. To diminish the potential confounding factor of hemolytic issues, only the first dish of collected follicular fluid which was collected at the beginning of fluid aspiration was included in the subsequent study. No additional flushing liquid was added to the fluid, and bloody samples were excluded from the study. The follicular fluid was centrifuged for 10 min at 3000 rpm to remove cell debris and other impurities. Approximately 500ul of the follicular fluid supernatant was ultimately collected and stored at -80 ℃ until the assay was performed [30].

### Assessment of AEs and AEEs

The concentrations of five AEs, namely Li, Na, K, Rb, and Cs, and four AEEs, including Mg, Ca, Sr, and Ba, in the follicular fluid were assessed. The remaining two elements, Fr and Ra, were not assessed because of their radioactivity. Be levels were not assessed because Be cannot be detected by the technology used in this study. Mass spectrometry was used to determine the concentrations of the target elements. Briefly, a 50 µL follicular fluid sample was preprocessed with 0.1 mL of the internal standard yttrium (0.2 µg/mL) and 4.85 mL of 1% nitric acid (ultrapure grade) as a mixture and K, Na, Ca, and Mg concentrations were analyzed with inductively coupled plasma emission spectrometry (iCap6000,, MA, USA). Another 100 µL of follicular fluid sample was preprocessed with 0.1 mL of mixed internal standard (indium and rhenium) and 1.8 mL of 1% nitric acid (ultrapure grade) as a mixture and Sr concentrations were analyzed with inductively coupled plasma-mass spectrometry (7700x, Agilent, Santa Clara, CA, USA). In addition, a 100 µL sample was preprocessed following the same procedure and Rb, Cs, and Ba concentrations were measured with inductively coupled plasma-mass spectrometry (Elan DRC II, PerkinElmer Sciex, Norwalk, CT, USA) [31].

During the assessment, several measures were taken to control for potential contamination. All of the Petri dishes and Eppendorf micro test tubes were sterilized to avoid potential element contamination. The machines and equipment were prescreened for contamination. In addition, a blank control (phosphate-buffered saline reagents that included an internal standard and nitric acid) was used and analyzed in the same way with each block of 15 samples to account for potential contamination in the preparation process. Clin-Chek®eControl Serum Control (National Laboratory of Nonferrous Metals and Electronic Materials Analysis and Testing Center, Beijing, China, Level II: 8881) was used as a quality control sample. In the final analysis, blank concentration was subtracted from the measured concentrations in each sample to determine the final concentration of each target element. Finally, the detection rates were calculated, and all the detection rates of the nine elements were 100% in our study (Table S1).

### Statistical analyses

Participant demographic characteristics were compared by chi-square tests or Fisher’s exact tests. Given that the levels of elements in our study were not normally distributed, their concentrations were described in terms of medians and interquartile ranges (25th percentile–75th percentile75, P25-P75), and the Mann–Whitney U test was used to compare the difference between groups. The *P* values were adjusted by Benjamini Hochberg’s multiple corrections. Spearman correlation analysis was used to explore the correlations between elements concentrations and dietary habits. Logistic regression was performed to assess associations between concentrations of individual elements and DOR by categorizing each element into high, middle, and low levels according to the tertile values for all subjects. Crude odds ratios (ORs) with 95% confidence intervals (CIs) were calculated. We adjusted for infertility type and female BMI and reported adjusted ORs as they were unevenly distributed between DORs and controls. To test the robustness of our results, we performed sensitivity analyses, which eatailed examining the associations between elements and DOR by categorizing the elements’ concentrations into three levels according to the tertiles of the control group.

Subsequently, the Bayesian kernel machine regression (BKMR) model was used to estimate the effect of coexposure to multiple elements on the likelihood of DORs. Logarithmic and Z-score transformations were performed on the concentrations of the elements. The mixture effect was estimated by BKMR analyses with hierarchical selection, which takes subgroups (AEs and AEEs) of the mixture into consideration [32]. The BKMR model was:

$$\eqalign{{\text{Yi}} & = h[{\text{groupAE}}({\text{Li}},{\text{Na}},{\text{K}},{\text{Rb}},{\text{Cs}}) \cr & \quad + {\text{groupAEE}}({\text{Mg}},{\text{Ca}},{\text{Sr}},{\text{Ba}})] \cr & \quad + \beta {\text{TZ}}i + ei. \cr}$$

The function *h* () was modeled using a Gaussian kernel exposure-response machine function, which allows for the inclusion of interaction terms. Z*i* refers to confounders (infertility type and BMI in this study), and e*i* represents residuals in the model. The β_probit_ in BKMR represents the estimated effect size of associations between exposure and outcome, which was transformed into a more straightforward indicator, OR, with the equation OR = exp (1.6 x β_probit_) [33, 34]. The BKMR analysis also enable the determination of the single effects for each specific element, defined as the change in the response associated with a change in a particular exposure from its 25th to its 75th percentile, where all of the other exposures are fixed at a specific quantile (0.25, 0.50, or 0.75); theunivariate exposure-response function, and the posterior inclusion probabilities (PIPs), whose values range from 0 to 1 and whose magnitude indicates relative variable importance.

R software (version 4.1.0, Austria), including the “BKMR” package for BKMR analysis and other R Core Teams packages, was used to perform the statistical analysis. The differences were considered statistically significant when the two-sided *P* value was < 0.05.

## Results

### Characteristics of participants

Table 1 shows that 308 participants (154 DOR and 154 controls) were involved in this study. The two groups showed significantly different frequencies between infertility types, and the DOR showed a higher rate of secondary infertility (27.9 vs. 17.5%, *P* = 0.041). BMI, was considered a confounder and was adjusted for in the subsequent association analysis. Other basic characteristics, such as age, education level, occupation, infertility duration, and indicators of thyroid function, were comparable between the groups. No women reported a history of smoking in our population, and only 13 participants reported drinking habits. The key markers of ovarian reserve, including AMH, FSH, and AFC were significantly different between the DOR patients and controls, indicating that the ovarian reserve was greater in the control than in the DOR group.

**Table 1:** Characteristics of women with diminished ovarian reserve (DOR) and controls in this study

Characteristics		DOR (*n* = 154)	Controls (*n* = 154)	P
**Female age, years** ^a^		30.0 (29.0–34.0)	29.5 (27.0–34.0)	0.117
**BMI [N(%)]**	< 18.5	8 (5.2)	13 (8.4)	0.032
	18.5–24.0	92 (59.7)	107 (69.5)	
	≥ 24.0	54 (35.1)	34 (22.1)	
**Infertility type [N(%)]**	Secondary	43 (27.9)	27 (17.5)	0.041
	Primary	111 (72.1)	127 (82.5)	
**Infertility duration, years [N(%)]**	< 5	100 (64.9)	93 (60.4)	0.192
	≥ 5	45 (29.2)	43 (27.9)	
	NA	9 (5.8)	18 (11.7)	
**Education [N(%)]**	Master degree or higher	20 (13.0)	21 (13.6)	0.734
	University/College	63 (40.9)	54 (35.1)	
	High school or lower	18 (11.7)	18 (11.7)	
	Unknown	53 (34.4)	61 (39.6)	
**Occupation [N(%)]**	Farmer	3 (1.9)	3 (1.9)	0.917
	Worker or officer	69 (44.8)	64 (41.6)	
	Free or others	82 (53.2)	87 (56.5)	
**Thyroid function**				
Free triiodothyronine (FT3) ^a^		3.45 (2.88–4.47)	3.37 (3.05–3.93)	0.650
Free thyroxine (FT4) ^a^		1.48 (1.31–9.50)	1.14 (1.08–4.94)	0.766
Thyrotropin (TSH) ^a^		2.04 (0.98–﻿3.00)	2.09 (1.48–4.09)	0.836
**Ovarian reserve function**				
Anti-mullerian hormone (AMH, ng/ml) ^a^		0.55 (0.22–0.74)	1.94 (1.89-2.00)	< 0.001
Follicle-stimulating hormone (FSH, IU/L) ^a^		10.43 (8.15–14.62)	8.24 (4.40–8.91)	< 0.001
Antral follicle count (AFC) ^a^		4.00 (2.25–﻿5.00)	10.50 (8.00–﻿11.50)	< 0.001

a The levels of the continuous variables were not normally distributed in the case and control group, so the Manney-Whitney test was used to compare the concentrations and the Median (P25-P75) was used to describe the levels of each compound

### Concentrations of AEs and AEEs in DOR patients and controls

Table 2 presents the concentrations of elements in the follicular fluid of DOR patients and controls. The median concentrations of Li (1.10 vs. 0.91 ng/ml), Na (3187.34 vs. 3130.50 µg/ml), and K (135.01 vs. 128.81 µg/ml) were greater in the DOR group (all adjusted *Ps* < 0.05). Although the differences were not statistically significant, the median levels of AEEs, including Mg, Ca, Ba, and Cs were relatively greater in the DOR group.

**Table 2:** Levels of alkali elements and alkaline earth elements in follicular fluid of cases and controls

	DOR (*n* = 154 )	Control (*n* = 154)	P^a^	AD-P
**Alkali elements**				
Li (ng/mL)	1.10 (0.74–1.89)	0.91 (0.61–1.33)	0.009	0.014
Na (ug/mL)	3187.34 (3055.05–﻿3266.11)	3130.50 (3027.02–﻿3235.35)	0.031	0.092
K (ug/mL)	135.01 (127.24–﻿141.61)	128.81 (122.55–﻿135.53)	< 0.001	< 0.001
Rb (ng/mL)	150.85 (131.25–171.70)	146.46 (131.50–﻿165.20)	0.420	0.511
Cs (ng/mL)	0.64 (0.51–0.78)	0.59 (0.48–0.69)	0.047	0.057
**Alkaline earth elements**				
Mg (ug/mL)	18.39 (17.11–20.58)	18.17 (17.00–﻿19.33)	0.095	0.170
Ca (ug/mL)	77.04 (72.93–86.72)	75.45 (72.78–87.74)	0.454	0.511
Sr (ng/mL)	38.83 (33.14–43.82)	40.04 (33.79–46.06)	0.238	0.104
Ba (ng/mL)	4.41 (2.96–7.31)	4.16 (2.87–6.73)	0.514	0.514

*Abbreviations* DOR, diminished ovarian reserve; Li, lithium; Na, sodium; K, potassium; Rb, rubidium; Cs, cesium; Mg, magnesium; Ca, calcium; Sr, strontium; Ba, barium. a: The concentrations of the elements were not normally distributed in the case and control group, so the Manney-Whitney test was used to compare the concentrations and the Median (P25-P75) was used to describe the levels of each compound. AD-P: adjusted P value by Benjamini Hochberg multiple corrections

### Associations between concentrations of individual elements and DOR: logistic regression

**Fig. 1:**
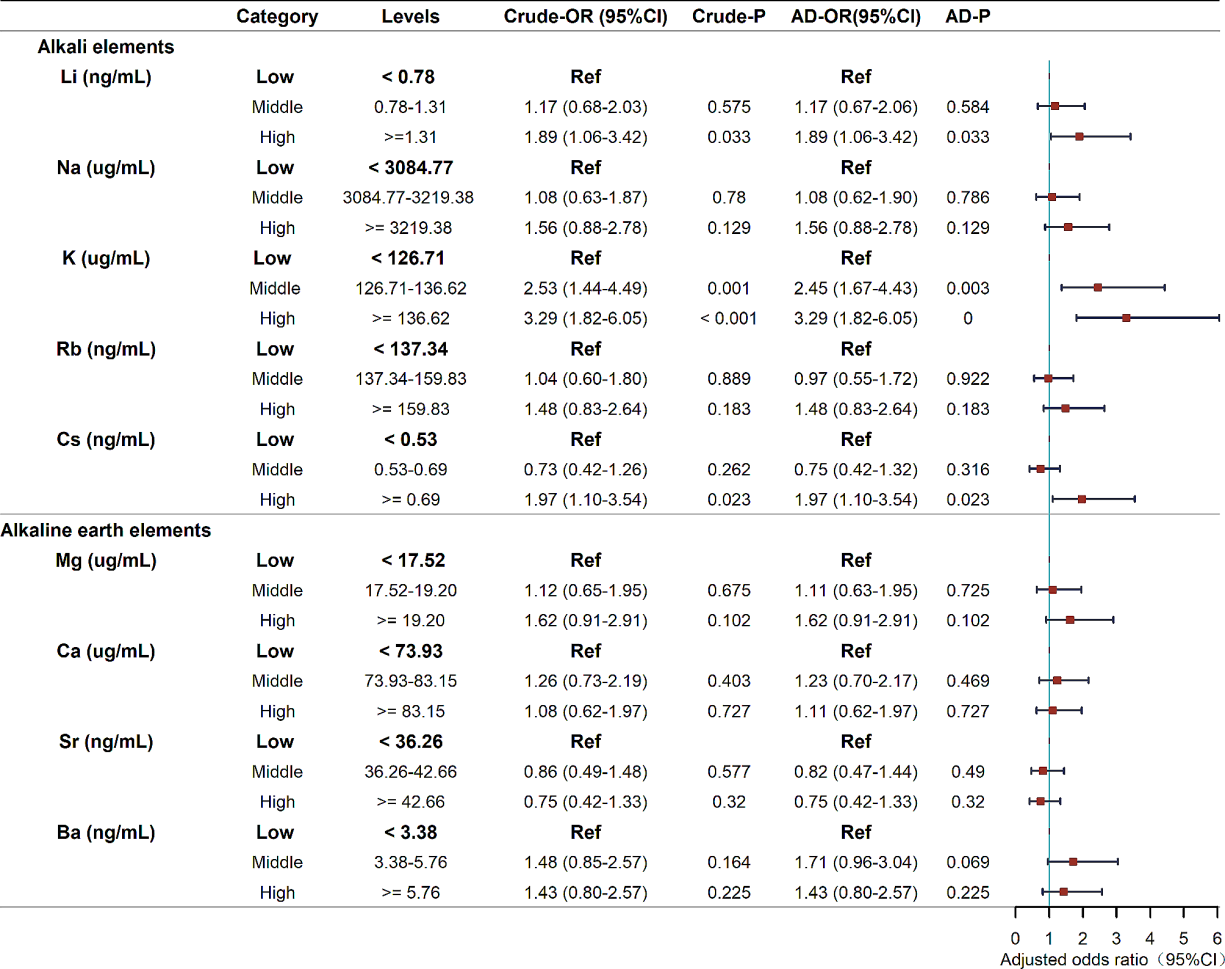
Associations between the levels of alkali elements and alkaline earth elements in follicular fluid and odds of a diminished ovarian reserve

As Fig. 1 shows, except for Sr, all remaining elements showed increased associations with the likelihood of DOR. The likelihood of DOR significantly increased with K concentration in a dose-dependent manner: compared with the low K level, the middle K level had an OR of 2.45 (1.67–4.43), while the high level had an OR of 3.29 (1.82–6.05). Although the middle levels of Li and Cs did not exhibit a statistically significant association with DOR, high levels of Li [OR: 1.89 (1.06–3.42)] and Cs [OR: 1.97 (1.10–3.54)] significantly increased the odds of DOR. A sensitivity analysis that categorized the concentrations of elements by the tertile of the control group showed that the associations were consistent with the results when the concentration was categorized by the tertiles of all participants (eTable 2).

### Associations between the concentrations of AE/AEE mixtures and DOR: BKMR analysis

**Fig. 2:**
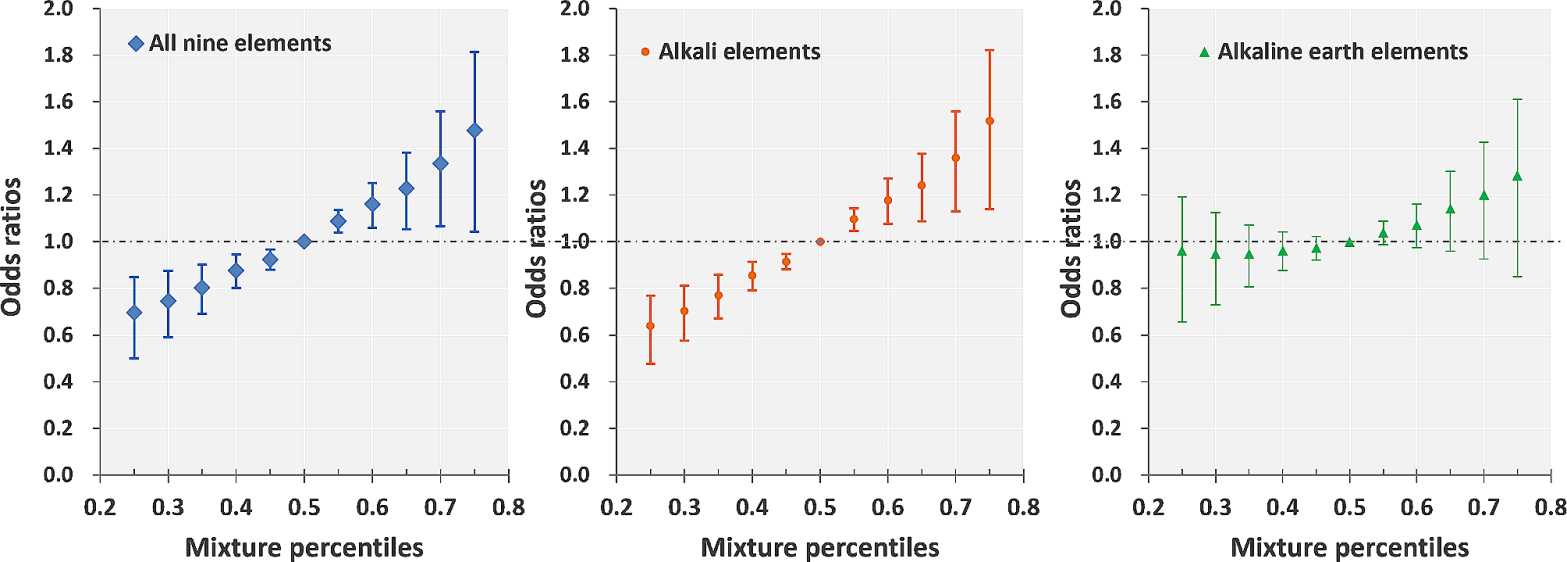
The overall effect of elements on the likelihood of a diminished ovarian reserve. Bayesian kernel machine regression was used to investigate the association between exposure to a mixture of 9 elements/5AEs/4 AEEs and DOR. The odds ratios were calculated where the mixture of elements was at a specific quantile level compared to when the mixture is at the 50th percentile. The estimated β-probit was transformed into an odds ratio using the formula OR = exp (1.6 x βprobit). The points refer to odds ratios (ORs), while the vertical lines represent the 95% CIs. The associations were adjusted for infertility type and BMI

The correlation analysis revealed that the concentrations of these AEs/AEEs have correlations in follicular fluid (eTable 3). Therefore, we used BKMR modeling, which enabled the combined effects of elements in a mixture on DOR to be analyzed. As Fig. 2 shows, the DOR increased linearly across the 25th percentile through the 75th percentile of the nine-AE/AEE mixture, with an OR of 1.48 (1.14–1.91) for the 75th percentile. Hierarchical analysis revealed that the mixed concentration of subgroup AEs (including Li, Na, K, Rb, and Cs) was significantly associated with a greater likelihood of DOR. The concentrations of AEEs (including Mg, Ca, Sr, and Ba) slightly increased with increasing DOR chance, but the difference was not statistically significant. The OR values for each percentile of the elements compared with the median on DOR are shown in eTable 4.

**Fig. 3:**
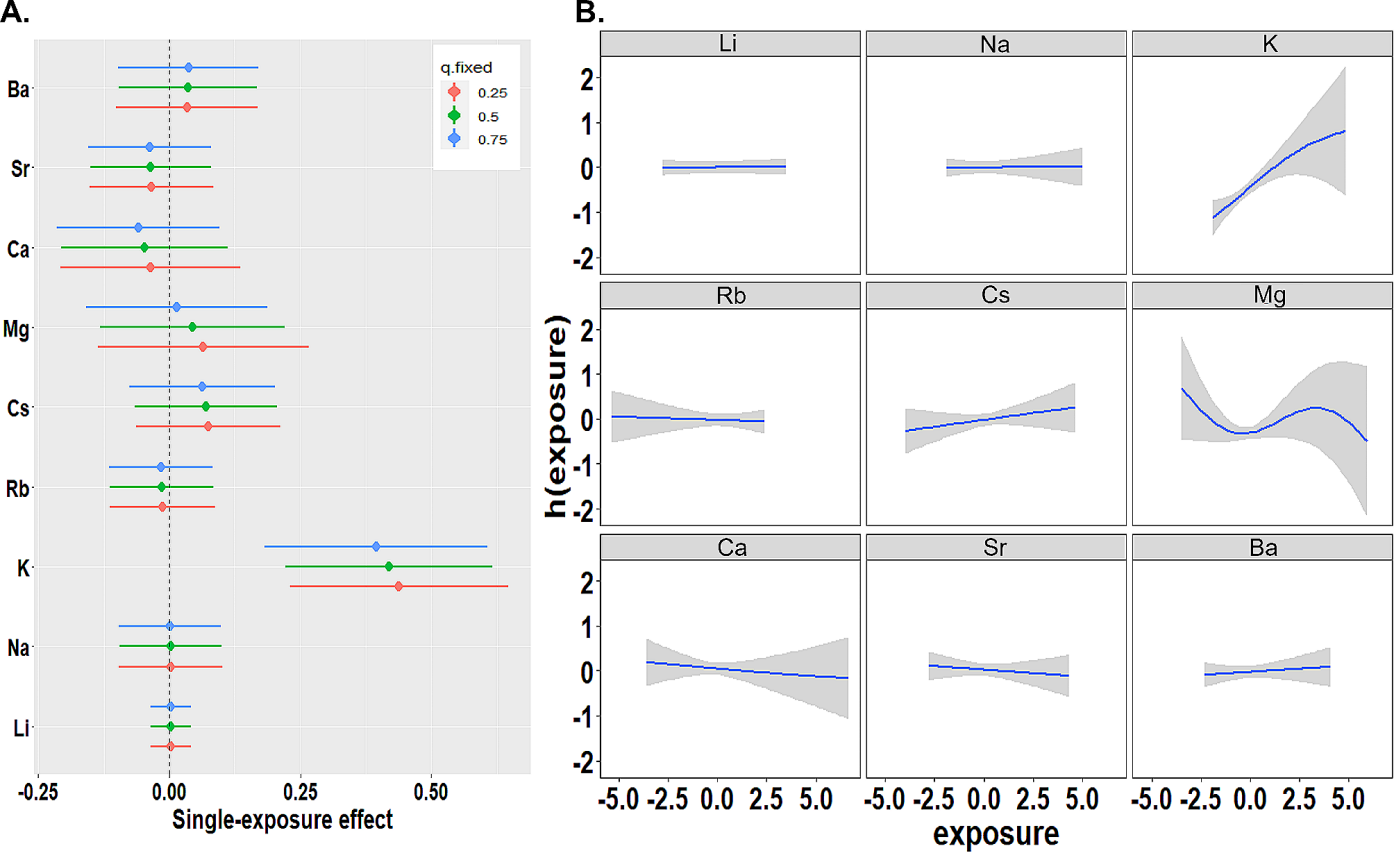
Associations between single element exposure and the likelihood of a diminished ovarian reserve according to the BKMR model: (**A**) This figure shows the estimated effect of a single element on the likelihood of DOR (est, expressed in βprobit) by comparing its 75th percentile to its 25th percentile, with all other elements being fixed at their 25th, 50th, or 75th percentile. The confounding factors adjusted for in this model include BMI and infertility type; (**B**) This figure shows the Univariate exposure–response function for each alkali element or alkaline earth element (95%CIs) with the remaining elements set at their medians. h(exposure) is defined as the association between a specific element as a continuous variable and a latent continuous outcome (continuous marker of the binary DOR outcome). The models were adjusted for infertility type and age. K, potassium; Na, sodium; Rb, rubidium; Cs, cesium; Ca, calcium; Mg, magnesium; Sr, strontium; Ba, barium

Figure 3a and eTable 5 show the single effects of elements on DOR in association with changes in specific elements from the 25th to 75th percentiles when other elements were fixed at the 25th, 50th, or 75th percentiles. K had the most potent effect on DOR, with effect estimates (βi) of 0.44 (95% CI: 0.23–0.65), 0.42 (95% CI: 0.22–0.62), and 0.40 (95% CI:0.18–0.61) when the remaining elements were kept at the 75th, 50th, and 25th percentiles, respectively. The PIPs, which were used to identify the elements or groups mainly responsible for DOR effects, validated the results. AEs showed higher group PIP than AEEs (0.97 vs. 0.54). Conditional PIPs for each element showed that among the nine AEs and AEEs, K had the highest PIP, indicating that K played the most critical role in the occurrence of DOR (**eFigure 1**). Figure 3b shows the dose-response curves when concentrations of an element were used as a continuous variable when all other elements were fixed at their 50th percentiles: K exhibited a positive linear trend, Mg showed an “S” trend, and no apparent trends were observed for the other elements. All of these results were mutually verified.

## Discussion

AEs and AEEs are among the most widely distributed elements essential to human biological processes. However, the effects of these factors on women’s fertility remain uncertain. In this study, we found that with the rising overall level of nine AE/AEE elements, the likelihood of DOR significantly increased in a linear trend. Specifically, compared with their lowest level, high exposure to K, Li, and Cs was significantly associated with an increased likelihood of DOR.

The balance of AEs and AEEs plays important roles in physiological functions [16]. Previous studies have indicated that AEs and AEEs are associated with female reproductive health [19, 23, 20–22]. However, at present, no studies have reported on the effect of AEs and AEEs on the ovarian reserve. This study provided new evidence that abnormal concentrations of several AE and AEEs could increase the likelihood of DOR. In addition, humans are more likely to be exposed a mixture of AEs and AEEs simultaneously, and elements have complex interactions. Therefore, assessing the combined effect of AE/AEE mixtures and taking the potential influence of other AEs/AEEs into consideration during the assessment of the likelihood posed by a specific element better reflects the reality of actual exposure dynamics [28, 31, 35]. At present, no studies have investigated the mixed impact of AE/AEEs on women’s fertility. For the first time, we observed that the odds for DOR significantly increased in a linear pattern with the higher concentration of the nine elements mixture. These results underlined the importance of considering the mixed effect of AEs and AEEs, especially AEs, in preventing DOR.

The potassium (K^+^) channel has important effects on reproductive health outcomes, and can be involved in outcomes such as malformation of the fallopian tube [36], and male infertility [37]. A population study reported that women with PCOS had a lower potassium intake than women in a control group [19]. Whether K plays a role in the ovarian reserve remains unclear. We found that a higher level of K in the follicular fluid increases the likelihood of DOR up to 3.29 fold, providing new evidence for the association between K and DOR. The accurate pathophysiology of K on DOR has yet to be determined. Previous studies have indicated that excessive potassium accumulation in the body and within cells leads to changes in membrane potential, which may be a contributing factor to the decline in fertility [38]. Additionally, it has been reported that intracellular potassium influx dysregulation is mediated by saturated fatty acids, leading to a series of adverse pathophysiological outcomes, destabilizing the pH of the cytosol, and thereby exacerbating the inflammatory response through activation of the arachidonic acid derivative cascade [39]. These studies may provide insight into the pathophysiology of K on DOR.

Li is a psycho-modulatory agent commonly used to treat depression, mania, schizophrenia, and other psychological disorders [40]. High doses of lithium in the human body cause the function of multiple key organs including the heart, thyroid gland, kidneys, and ovarians, to deteriorate [41]. A population study in China reported that exposure to Li could decrease the hormone testosterone, which is associated with male infertility [42]. It has been reported that mechanistically, both the positive and negative effects of lithium could be mediated through the methylation of β-catenin nuclear-binding proteins which is potentiated by lithium-induced inhibition of GSK-3 or inositol monophosphatase [43]. Animal studies have shown that the concentration of lithium leads to follicular atresia and this adverse effect has been found to result from the induction of apoptosis in antral follicles [44]. The chemical properties of Cs are similar to those of potassium, enabling it to displance potassium in muscles and red blood cells, thus causing potassium deficiency [45]. However, the effects of Li and Cs on the female ovarian reserve are unknown. In this study, we found that higher levels of Li and Cs could increase the likelihood of DOR. In contrast, the middle levels of these two elements did not show a statistically significant effect, indicating that within limitations, Li or Cs might not show significant toxicity, but they would influence ovarian reserve function when their levels exceeded certain limits.

Mg and Ca are ubiquitous and essential to living organisms. The associations between Mg/Ca and female infertility are uncertain [46, 47]. Sr, another crucial AEE, was reported to be associated with DOR progression [OR: 2·92 (1·86 − 4·58)] [48], and Ba was reported to be associated with an increased risk of PCOS [23] and early embryonic arrest [49]. In our study, we found that although the concentrations of each AEE were relatively greater in DOR patients than in controls, their individual effects on DOR were not significant, implying that the association between AEEs and DOR might require further validation.

There are several strengths in this study. First, previous studies often used serum or urine samples to assess the environmental factors of ovarian reserve. However, for oocytes, their development and maturation occur within a barrier environment composed of follicular fluid formed by granulosa cells and plasma ultrafiltrate, without direct contact with the bloodstream. Therefore, it has been hypothesized that elements detected in follicular fluid may directly affect the development and maturation of oocytes, and recent studies have used ovarian follicular fluid to provide a more accurate estimate of exposures that may influence reproductive outcomes. Therefore, in this study, we assessed the levels of elements in follicular fluid rather than in blood to determine whether the concentrations of these elements accurately reflected the direct exposure status of oocytes. Second, to our knowledge, our study is the first to report the combined effect of AEs and AEEs in follicular fluid on ovarian reserve function and to identify important contributors to the impact of ovarian reserve. Third, considering the critical influence of women’s age on DOR, we controlled for age in this study to eliminate the confounding effect of age. In addition, the postpowers of the sample size in this study based on each element ranged from 89 to 98%, indicating that we included a sufficient sample size to reach high testing efficiency.

On the other hand, some limitations need to be addressed. First, since our study was based on a case-control design, our study only provided an initial clue for the association between AEs/AEEs and DOR. The causal correlation between these elements and the occurrence of DOR requires further validation in animal models or prospective human studies. Second, the biological mechanism underlying the associations between identified AEs and AEEs and DOR was uncertain. Further investigations in animals and in vitro studies are needed. Third, in our study, medical intake information was unavailable, which may let us to fail to account for the medical source of AEs/AEEs. Finally, although we focused on the content of AE/AEEs in the follicular fluid, which directly interacts with oocytes, we did not measure the concentration of these elements in the blood or provide a detailed analysis of their sources. This limitation somewhat constrained the process of causal inference.

## Conclusion

This study revealed an association in which the likelihood of DOR increased with higher overall concentrations of AE/AEEs in follicular fluid. Among the nine detected elements, the higher concentrations of K, Li, and Cs in follicular fluid exhibited a significant positive correlation with the likelihood of DOR. These findings provide novel evidence for the potential environmental factors of female fertility decline. These findings warrant further investigation and replication in additional studies.

### Electronic supplementary material

Below is the link to the electronic supplementary material.

Supplementary Material 1

## Data Availability

The data underlying this article will be shared on reasonable request to the corresponding author.
