# Supplementary material for: Alkali and alkaline earth elements in follicular fluid and the likelihood of diminished ovarian reserve in reproductive-aged women: a case‒control study
Source: J Ovarian Res. 2024 May 18;17:108. doi: 10.1186/s13048-024-01414-3 (PMC11102265; doi:10.1186/s13048-024-01414-3)

**eTable 1. Information of the detected elements in this study**

| **Element** | **Abbreviation** | **Limit of detection (ng/ml)** | **Detection rate (%)** |
| --- | --- | --- | --- |
| Lithium | Li | 0.54 | 100 |
| Sodium | Na | 195.90 | 100 |
| Potassium | K | 200.00 | 100 |
| Rubidium | Rb | 0.08 | 100 |
| Cesium | Cs | 0.02 | 100 |
|  |  |  |  |
| Magnesium | Mg | 200.00 | 100 |
| Calcium | Ca |  | 100 |
| Strontium | Sr | 0.20 | 100 |
| Barium | Ba | 0.04 | 100 |

**eTable 2. Associations between levels of alkali elements and alkaline earth elements follicular fluid and odds for diminished ovarian reserve: (Concentrations were categorized by the tertile of the control group)**

|  | **Category** | **Levels** | **Crude-OR (95%CI)** | **Crude-P** | **AD-OR**  **(95%CI)** | **AD-P** |
| --- | --- | --- | --- | --- | --- | --- |
| Alkali elements | |  |  |  |  |  |
| Li (ng/mL) | Low | < 0.72 | Ref |  | Ref |  |
|  | Middle | 0.72-1.20 | 1.37 (0.76-2.47) | 0.293 | 1.39 (0.77-2.55) | 0.276 |
|  | High | ≥1.20 | 1.98 (1.14-3.47) | 0.016 | 1.91 (1.08-3.41) | 0.027 |
| Na (ug/mL) | Low | < 3074.66 | Ref |  | Ref |  |
|  | Middle | 3074.66-3198.35 | 0.82 (0.45-1.46) | 0.489 | 0.76 (0.42-1.38) | 0.374 |
|  | High | ≥ 3198.35 | 1.60 (0.94-2.77) | 0.087 | 1.59 (0.91-2.77) | 0.103 |
| K (ug/mL) | Low | < 123.80 | Ref |  | Ref |  |
|  | Middle | 123.80-133.08 | 2.55 (1.35-4.93) | 0.004 | 2.69 (1.39-5.35) | 0.004 |
|  | High | ≥ 133.08 | 4.10 (2.23-7.79) | < 0.001 | 4.31 (2.28-8.41) | < 0.001 |
| Rb (ng/mL) | Low | < 136.82 | Ref |  | Ref |  |
|  | Middle | 136.82-158.69 | 0.96 (0.55-1.68) | 0.887 | 0.88 (0.49-1.54) | 0.629 |
|  | High | ≥ 158.69 | 1.23 (0.71-2.12) | 0.457 | 1.29 (0.74-2.26) | 0.377 |
| Cs (ng/mL) | Low | < 0.51 | Ref |  | Ref |  |
|  | Middle | 0.51-0.65 | 0.92 (0.51-1.67) | 0.791 | 0.87 (0.47-1.59) | 0.65 |
|  | High | ≥ 0.65 | 1.73 (1.01-3.10) | 0.044 | 1.68 (0.96-2.96) | 0.069 |
| Alkaline earth elements | |  |  |  |  |  |
| Mg (ug/mL) | Low | < 17.36 | Ref |  | Ref |  |
|  | Middle | 17.36-18.99 | 0.96 (0.54-1.69) | 0.885 | 1.11 (0.63-1.95) | 0.998 |
|  | High | ≥ 18.99 | 1.44 (0.84-2.49) | 0.185 | 1.62 (0.91-2.91) | 0.209 |
| Ca (ug/mL) | Low | < 73.38 | Ref |  | Ref |  |
|  | Middle | 73.38-81.27 | 1.40 (0.81-2.44) | 0.231 | 1.23 (0.70-2.17) | 0.231 |
|  | High | ≥ 81.27 | 1.24 (0.71-2.18) | 0.456 | 1.11 (0.62-1.97) | 0.469 |
| Sr (ng/mL) | Low | < 36.67 | Ref |  | Ref |  |
|  | Middle | 36.67-43.65 | 0.95 (0.56-1.61) | 0.841 | 0.93 (0.54-1.61) | 0.807 |
|  | High | ≥ 43.65 | 0.69 (0.39-1.21) | 0.193 | 0.70 (0.39-1.22) | 0.203 |
| Ba (ng/mL) | Low | < 3.21 | Ref |  | Ref |  |
|  | Middle | 3.21-5.65 | 1.23 (0.71-2.15) | 0.463 | 1.42 (0.80-2.55) | 0.229 |
|  | High | ≥ 5.65 | 1.33 (0.76-2.31) | 0.319 | 1.47 (0.83-2.62) | 0.189 |

**eTable 3. Correlations between concentrations of alkali elements and alkaline earth elements follicular fluid**

|  |  | **Li** | **Na** | **K** | **Rb** | **Cs** | **Mg** | **Ca** | **Sr** | **Ba** |
| --- | --- | --- | --- | --- | --- | --- | --- | --- | --- | --- |
| Li | r | 1.000 | .239^**^ | .357^**^ | -0.007 | .128^*^ | .332^**^ | .159^**^ | 0.056 | 0.031 |
| Na | r |  | 1.000 | .364^**^ | .240^**^ | .222^**^ | .467^**^ | .112^*^ | 0.090 | -.123^*^ |
| K | r |  |  | 1.000 | .154^**^ | .219^**^ | .369^**^ | .316^**^ | 0.014 | 0.049 |
| Rb | r |  |  |  | 1.000 | .690^**^ | .139^*^ | 0.090 | 0.016 | -0.055 |
| Cs | r |  |  |  |  | 1.000 | .154^**^ | .176^**^ | 0.006 | -.125^*^ |
| Mg | r |  |  |  |  |  | 1.000 | .388^**^ | -0.073 | -.122^*^ |
| Ca | r |  |  |  |  |  |  | 1.000 | -0.070 | 0.094 |
| Sr | r |  |  |  |  |  |  |  | 1.000 | .135^*^ |
| Ba | r |  |  |  |  |  |  |  |  | 1.000 |

*. Correlation is significant at the 0.05 level (2-tailed). **.*. Correlation is significant at the 0.001 level (2-tailed)

**eTable 4. Odds ratio values in BKMR model**

| Quantile | **All nine elements** | | |  | **Alkali elements** | | |  | **Alkaline earth elements** | | |
| --- | --- | --- | --- | --- | --- | --- | --- | --- | --- | --- | --- |
|  | OR | Lower-OR | Upper-OR |  | OR | Lower-OR | Upper-OR |  | OR | Lower-OR | Upper-OR |
| 0.25 | 0.70 | 0.54 | 0.89 |  | 0.64 | 0.51 | 0.80 |  | 0.96 | 0.73 | 1.27 |
| 0.30 | 0.75 | 0.62 | 0.90 |  | 0.70 | 0.60 | 0.83 |  | 0.95 | 0.77 | 1.17 |
| 0.35 | 0.80 | 0.70 | 0.92 |  | 0.77 | 0.68 | 0.87 |  | 0.95 | 0.82 | 1.09 |
| 0.40 | 0.88 | 0.81 | 0.95 |  | 0.85 | 0.80 | 0.92 |  | 0.96 | 0.88 | 1.05 |
| 0.45 | 0.92 | 0.88 | 0.97 |  | 0.92 | 0.88 | 0.95 |  | 0.97 | 0.93 | 1.03 |
| 0.50 | 1.00 | 1.00 | 1.00 |  | 1.00 | 1.00 | 1.00 |  | 1.00 | 1.00 | 1.00 |
| 0.55 | 1.09 | 1.04 | 1.14 |  | 1.10 | 1.05 | 1.15 |  | 1.04 | 0.99 | 1.09 |
| 0.60 | 1.16 | 1.07 | 1.26 |  | 1.18 | 1.08 | 1.28 |  | 1.07 | 0.98 | 1.17 |
| 0.65 | 1.23 | 1.08 | 1.40 |  | 1.24 | 1.10 | 1.39 |  | 1.14 | 0.98 | 1.33 |
| 0.70 | 1.34 | 1.11 | 1.60 |  | 1.36 | 1.16 | 1.59 |  | 1.20 | 0.98 | 1.48 |
| 0.75 | 1.48 | 1.14 | 1.91 |  | 1.52 | 1.22 | 1.90 |  | 1.28 | 0.96 | 1.72 |

**eTable 5. Estimate of single effects in BKMR model**

| **Percentile** | **Element** | **βi** | **95% CI of βi** | |
| --- | --- | --- | --- | --- |
|  |  |  | **Lower** | **Upper** |
| 0.25 | Li | 0.003 | -0.036 | 0.042 |
| 0.25 | Na | 0.003 | -0.095 | 0.101 |
| 0.25 | K | 0.439 | 0.231 | 0.646 |
| 0.25 | Rb | -0.013 | -0.113 | 0.087 |
| 0.25 | Cs | 0.075 | -0.062 | 0.212 |
| 0.25 | Mg | 0.065 | -0.136 | 0.267 |
| 0.25 | Ca | -0.036 | -0.207 | 0.136 |
| 0.25 | Sr | -0.034 | -0.152 | 0.085 |
| 0.25 | Ba | 0.034 | -0.101 | 0.170 |
| 0.5 | Li | 0.003 | -0.036 | 0.042 |
| 0.5 | Na | 0.003 | -0.095 | 0.100 |
| 0.5 | K | 0.419 | 0.221 | 0.618 |
| 0.5 | Rb | -0.014 | -0.113 | 0.085 |
| 0.5 | Cs | 0.070 | -0.065 | 0.206 |
| 0.5 | Mg | 0.045 | -0.131 | 0.220 |
| 0.5 | Ca | -0.047 | -0.206 | 0.112 |
| 0.5 | Sr | -0.035 | -0.150 | 0.080 |
| 0.5 | Ba | 0.036 | -0.095 | 0.167 |
| 0.75 | Li | 0.003 | -0.036 | 0.042 |
| 0.75 | Na | 0.002 | -0.095 | 0.099 |
| 0.75 | K | 0.395 | 0.182 | 0.607 |
| 0.75 | Rb | -0.015 | -0.114 | 0.084 |
| 0.75 | Cs | 0.063 | -0.076 | 0.203 |
| 0.75 | Mg | 0.014 | -0.159 | 0.187 |
| 0.75 | Ca | -0.059 | -0.214 | 0.096 |
| 0.75 | Sr | -0.037 | -0.154 | 0.080 |
| 0.75 | Ba | 0.037 | -0.097 | 0.171 |

**eTable 6. Correlations between elements levels and diet frequencies**

|  | **Fish** | **Meat** | **Eggs** | **Milk** | **Fresh vegetables** | **Fresh fruits** | **Beans or bean product** | **Barbecue food** | **Pickled foods** |
| --- | --- | --- | --- | --- | --- | --- | --- | --- | --- |
| Li | 0.068 | 0.125 | 0.013 | 0.061 | 0.100 | 0.110 | 0.058 | 0.009 | 0.066 |
| Na | -0.017 | 0.121 | 0.126 | 0.024 | 0.051 | 0.144* | -0.038 | 0.050 | 0.064 |
| K | -0.032 | 0.053 | 0.046 | 0.039 | 0.021 | 0.045 | -0.079 | 0.009 | 0.057 |
| Rb | -0.069 | 0.087 | 0.134 | 0.269* | 0.053 | 0.037 | 0.011 | 0.025 | -0.136 |
| Cs | -0.02 | 0.127 | 0.059 | 0.276* | 0.077 | -0.003 | 0.001 | -0.004 | -0.075 |
| Mg | 0.045 | 0.077 | 0.091 | -0.015 | -0.021 | 0.114 | -0.029 | 0.004 | 0.085 |
| Ca | 0.004 | 0.077 | 0.127 | 0.089 | -0.037 | 0.065 | -0.016 | -0.104 | 0.005 |
| Sr | -0.009 | 0.097 | -0.046 | -0.218 | -0.028 | 0.023 | -0.029 | 0.031 | -0.059 |
| Ba | 0.087 | 0.039 | -0.013 | -0.103 | -0.014 | -0.096 | 0.053 | 0.145* | 0.022 |

*. Correlation is significant at the 0.05 level (2-tailed).

**eFigure 1.Group and conditional posterior inclusion probabilities (PIPs) of alkali elements and alkaline earth elements**


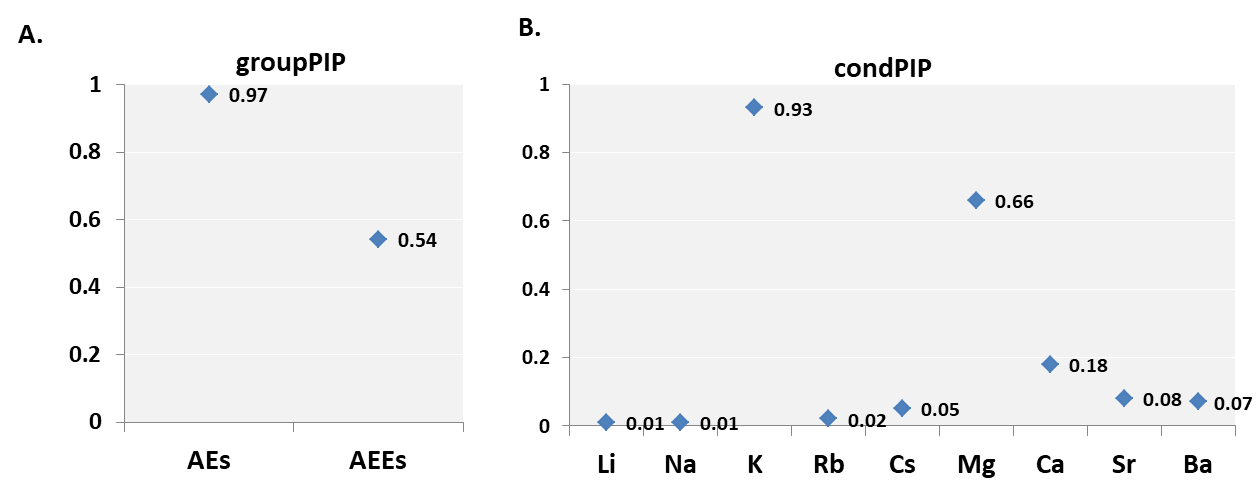

Supplement: Supplementary file 1 — Supplementary Material 1 [file 13048_2024_1414_MOESM1_ESM.docx]
